# Supplementary material for: Insertion Hot Spots of DIRS1 Retrotransposon and Chromosomal Diversifications among the Antarctic Teleosts Nototheniidae
Source: Int J Mol Sci. 2019 Feb 6;20(3):701. doi: 10.3390/ijms20030701 (PMC6387122; doi:10.3390/ijms20030701)
Supplement: Supplementary file 1 [file ijms-20-00701-s001.zip › ijms-426464-supplementary.pdf]

International Journal of *Molecular Sciences*

Insertion Hot Spots of *DIRS1* Retrotransposon and Chromosomal Diversifications Among the Antarctic Teleosts Nototheniidae

Supplementary materials

Table S1. TE intrafamily conservation for *YNoto*, *GyNoto*, and *CoNoto* sequences within and across distinct nototheniid sub-families. Complement of Table 1. Nucleotide identity percentages.

YNoto B-R-J-V	Trem	Pleu	Noto	Diss	Chan	Cygn	Gymn	Arte	Gobi
Trem	93-95-93-92	85-x-85-91	86-87-88-87	x-90-79-89	87-86-89-89	87-89-90-x	x-88-x-91	88-89-89-87	x-x-89-89
Pleu		x-x-x-x	87-x-88-87	x-x-76-90	85-x-84-89	88-x-85-x	x-x-x-90	86-x-84-89	x-x-84-87
Noto			87-91-88-84	x-94-86-87	84-88-87-86	85-91-89-x	x-90-x-85	86-92-90-89	x-x-90-89
Diss				x-x-x-x	x-89-78-88	x-92-80-x	91-x-88-x	x-93-78-89	x-x-78-86
Chan					x-x-87-86	91-93-90-x	x-92-x-89	95-94-89-90	x-x-88-85
Cygn						x-x-x-x	x-95-x-x	93-96-91-x	x-x-91-x
Gymn							x-x-x-x	x-98-x-86	x-x-x-86
Arte								X-95-95-97	x-x-93-86
Gobi									x-x-x-x

The first table represents *YNoto* in the following order: *YNotoB, R, J, V*.

GyNoto A-B-E-F-H	Trem	Pleu	Noto	Diss	Chan	Cygn	Gymn	Arte	Gobi
Trem	96/96-86/86-97/97-97-97	x/x-90/90-x/x-x-x	95/90-86/88-94/94-94-95	94/93-87/86-95/97-96-89	93/94-88/86-95/96-96-92	94/91-90/x-94/96-96-95	x/x-85/84-96/95-95-95	95/95-86-95/96-95-94	93/92-91/89-x/x-x-x-
Pleu		x/x-x/x-x/x-x-x	x/x-85/92-x/x-x-x	x/x-84/85-x/x-x-x	x/x-89/89-x/x-x-x	x/x-95/x-x/x-x-x	x/x-82/82-x/x-x-x	x/x-83-x/x-x-x	x/x-96/97-x/x-x-x
Noto			x/88-83/88-94/94-94-95	95/90-87/85-93/94-94-89	94/91-87/87-95/95-95-93	94/87-86/x-94/95-95-96	x/x-87/84-95-94-94-96	97/92-87-95/95-94-95	94/89-87/92-x/x-x-x
Diss				x/x-x/x-x/x-x-x	94/93-88/85-94/96-96-91	97/87-84/x-94/96-97-89	x/x-91-89-95/96-96-89	95/95-91-94/96-95-88	93/93-85/84-x/x-x-x-x
Chan					95/97-83/83-97/98-99-92	92/94-89/x-96/97-98-94	x/x-88/84-97/96-97-94	95/97-88-96/97-97-93	93/93-90/89-x/x-x-x
Cygn						x/x-x/x-x/x-x-x	x/x-83/x-96/96-97-98	94/92-84/x-95/97-97-97	92/90-96/x-x/x-x-x
Gymn							x/x-x/x-x/x-x-x	x/x-97-97/96-96-97	x/x-84/82-x/x-x-x
Arte								99/98-x/x-x/x-x-96	94/94-84/85-96/97-x-x
Gobi									x/x-x/x-x/x-x-x

The second table represents the *GyNoto* in the following order *GyNotoA, B, E, F, H*.

CoNoto A-B	Trem	Pleu	Noto	Diss	Chan	Cygn	Gymn	Arte	Gobi
Trem	x-96	x-95	x-96	x-92	x-x	x-x	x-97	x-88	x-x
Pleu		x-x	x-96	x-92	x-x	x-x	x-97	x-88	x-x
Noto			98-96	98-93	97-x	x-x	98-97	99-90	98-x
Diss				x-x	97-x	x- x	98-93	99-87	98-x
Chan					96-x	x-x	97-x	97-x	96-x
Cygn						x-x	x-x	x-x	x-x
Gymn							x-x	98-90	98-x
Arte								x-x	99-x
Gobi									x-x

The third table represent the *CoNoto* in the following order: *CoNotoA* and *B*. RT/RH and Int portions of the *Gypsy* sequences were integrated in the same table for *GyNotoA*, *B*, and *E*. *GyNotoF* and *H* are spanning the Int portion. X: not applicable (when only one specimen per species or when specimen were removed because of alignment difficulties).

	Intra-clade mean	Inter-clade mean
DIRS1	90.7	88.3
Gypsy (RT-RH)	92.4	90.6
Gypsy (Int)	94.3	91.5
Gypsy total	93.5	91.1
Copia GalEa	97.0	97.8
Copia Hydra	96.0	95.6

The fourth table represents the intra vs inter-clade (nototheniid sub-family) mean identity percentages for each TE are presented in the last table.

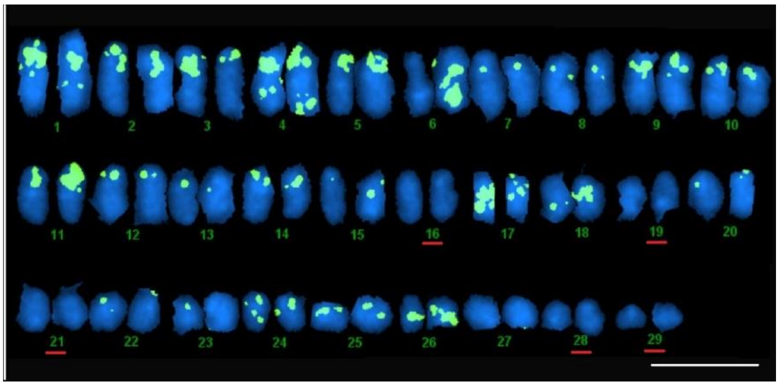

Figure S2. *YNotoJ* localized on chromosomes of *T. nicolai* (2n = 58). *DIRS1* probe (corresponding to the *YNotoJ* family) was labeled with biotin and bound probe was detected with incubation with Avidin-FITC (fluorescein, greenish spots). Probe characteristics are indicated in Additional file 4. Chromosomal DNA was counterstained with DAPI. *DIRS1* forms multiple insertion hot spots preferentially located in centromeric/pericentromeric regions (giving a type 1 insertion pattern). The five chromosomal pairs where no *DIRS1* were observed are underlined in red. Scale bars: 10 μm.

Table S2. Taxonomic sampling for tissues and chromosomal preparations used in this study.

sub-family	*Genus. Species*	Sampling type	Field reference	Locality and voucher reference (when applicable)
Trematominae	*T. eulepidotus*	tissue	si487n3152[5]	Adelie Bank, Adelie Land margin, MNHN 2009-1358
		chromosomes	CE2381[7]	
	T. nicolai	tissue	TNB214[1]	Adelie Bank, Adelie Land margin
		chromosomes	CE5684REVO856[7]	
	T. bernacchii	tissue	si352n2561[5]<br>TA337TRBE7[4]	Adelie Bank, Adelie Land margin, MNHN 2009-1312
		chromosomes	REVO2009-10 214-2738[7]	
	L. nudifrons	tissue	ICEFISH2005SR[2]	Shag rocks, South Atlantic
	L. larseni	tissue	ICEFISH2004SR[2]	Shag rocks, South Atlantic
	L. squamifrons	tissue	ST59N°1249[2]	Southern Shetlands, SAIAB 74977
	P. guentheri	tissue	ICEFISH2004[2]	South Georgia
Pleuragramminae	*P. antarctica*	tissue	si394n2707[5]	Adelie Basin, Adelie Land margin, MNHN 2009-1332
Nototheniinae	*N. coriiceps*	tissue	TA421NOCO8[4]	Adelie Bank, Adelie Land margin, MNHN 2008-1884
		chromosomes	TA449[4]	
	N. angustata	tissue	Nang1	Southern New Zealand
	N. rossi	tissue	ICEFISH2004SG[2]	South Georgia
	P. magellanica	tissue	Aus29[6]	Kerguelen Plateau, MNHN 2007-1843
Dissostichinae	*D. mawsoni*	tissue	REVO759[7]	Adelie Bank, Adelie Land margin, MNHN 2001-1143
Channichthyinae	*C. hamatus*	tissue	TNB235[1]	Terra Nova Bay
		chromosomes	R60-II[0]	
	C. antarcticus	tissue	TNB246[1]	Terra Nova Bay
	C. gunnari	tissue	ICEFISH2004SR[2]	Shag rocks, South Atlantic
	C. esox	tissue	ICEFISH2004F[2]	Falkland Islands
Cygnodraconinae	*C. mawsoni*	tissue	TA53CYMA1[4]	Adelie Basin, Adelie Land margin
Gymnodraconinae	*G. acuticeps*	tissue	TA109GYAC1[4]	Adelie Bank, Adelie Land margin
Artedidraconinae	*P. scotti*	tissue	si238n1819[5]	Mertz Bank, George V land coast, MNHN 2009-1389
	H. velifer	tissue	si283n978[5]	Adelie Bank, George V land margin/Coulman Island, MNHN 2009-0967[4]
		chromosomes	VLT44[3]	
Gobionototheniinae	*G. gibberifrons*	tissue	ICEFISH2004SR[2]	South Georgia

Tissue samples and chromosomal preparations were collected during the French, Italian, American, and International Antarctic campaigns: **TNB**[0] (1990/1991, Mario Zuchelli Sation, Ross sea), **I.C.E.FISH**[1] (1999, Zuchelli Station, Ross sea), **ICE FISH**[2] (2004, Atlantic part of the Southern Ocean), **VLT**[3] (Victoria Land Transect, 2004, Ross Sea), **ICOTA**[4] (1996-2008, Adelie Land), **CEAMARC**[5] (2007/2008 north of Adélie Land and George V Land, Eastern Antarctica), **POKER**[6] (2010 and 2013 Kerguelen-Heard shelf), **REVOLTA**[7] (2010-2014, Adélie Land).TNB235, TNB246, TNB214, R60-II, VLT44 were collected by Eva Pisano in Ross sea and conserved in the National Italian Antarctic Museum (no voucher reference). Nang1 was collected by Chris Cheng in Southern New Zealand (no voucher reference). A single voucher reference is provided per species/locality. Other specimens are deposited in the MNHN research collections.

Table S3. Summary of TE probes used for FISH. For precisions (origin, survey), see Table S2 (taxonomic sampling).

TEs	Family	Sequence name	Species related	N° specimen for probes	Insert size (kb)
DIRS1	*YNotoJ*	*YChaJ*	*C. hamatus*	TNB235	1.00
	YNotoJ	*YHveJ*	*H. velifer*	si283n978	1.06
	YNotoJ	*YGacuJ*	*G. acuticeps*	TA109	1.19
	YNotoJ	*YTbeJ*	*T. bernacchii*	TA337TRBE7	1.10
Gypsy	*GyNotoA*	*GyGacA*	*G. acuticeps*	TA109	1.54
	GyNotoA	*GyChaA*	*C. hamatus*	TNB235	1.53
	GyNotoA	*GyHveA*	*H. velifer*	si283n978	1.43
Copia	*CoNotoA* (*GalEa*)	*CoGacA*	*G. acuticeps*	TA109	0.94
	CoNotoA (GalEa)	*CoPscA*	*P. scotti*	si238n1819	0.93
	CoNotoB (Hydra)	*CoHveB*	*H. velifer*	si283n978	1.37
	CoNotoB (Hydra)	*CoGacuB*	*G. acuticeps*	TA109	1.37
